# Integrative multi-transcriptomics and network pharmacology reveal natural therapeutics as anti-cancer agents targeting AURKA for ovarian cancer treatment

**DOI:** 10.1016/j.jgeb.2026.100745

**Published:** 2026-06-20

**Authors:** Hriddhi Sarker, Md. Ahad Ali, Md Fakhrul Islam, Enam Ahmed, Amlan Ganguly, Md. Nazmul Hasan Zilani

**Affiliations:** a Department of Biochemistry & Molecular Biology, University of Rajshahi, Rajshahi 6205, Bangladesh; b Department of Computational Chemistry and Drug Design, Panacea Research Center, Rajshahi 6206, Bangladesh; c Bioinformatics Lab, Department of Statistics, University of Rajshahi, Rajshahi 6205, Bangladesh; d Department of Food Engineering and Tea Technology, Shahjalal University of Science and Technology, Kumargaon, Sylhet 3114, Bangladesh; e Department of Clinical Pharmacy and Pharmacology, Faculty of Pharmacy, University of Dhaka, Dhaka 1000, Bangladesh; f Department of Pharmacy, Faculty of Biological Science and Technology, Jashore University of Science and Technology, Jashore 7408, Bangladesh

**Keywords:** Ovarian Cancer, Network pharmacology, Pan-cancer analysis, Molecular docking, MD simulation

## Abstract

Ovarian cancer (OC) remains one of the most lethal gynecologic malignancies, largely because it is often diagnosed late, recurs frequently, and develops resistance to conventional chemotherapy. These challenges emphasize the urgent need for novel and less toxic therapeutic strategies. In this study, we applied an integrative in silico workflow combining transcriptomic analysis, network biology, pan-cancer exploration, and computational drug discovery to identify potential phytochemical inhibitors targeting Aurora kinase A (AURKA) in OC. Differential gene expression analysis of three GEO datasets (GSE14407, GSE18520, and GSE26712) identified common dysregulated genes associated with OC progression. Functional enrichment and regulatory network analyses revealed strong involvement of these genes in cell-cycle regulation, mitotic progression, and cancer-related signaling pathways. Protein–protein interaction analysis identified AURKA as a central hub gene with high network connectivity and strong overexpression across multiple cancer types. Further pan-cancer analyses demonstrated significant associations of AURKA with immune infiltration patterns and recurrent genetic alterations, supporting its therapeutic relevance. After removing duplicates from the 6834 collected phytochemicals, a total of 2046 unique compounds derived from medicinal plants of the Indian subcontinent were screened against the AURKA crystal structure using molecular docking to identify potential inhibitors. Several compounds demonstrated binding affinities comparable to the reference inhibitor Y3M. Subsequent drug-likeness, ADMET, toxicity prediction, molecular dynamics simulation, MM-GBSA binding energy analysis, and quantum mechanical evaluations identified CID-87014 (2-(Hydroxymethyl)anthraquinone) as the most promising candidate. This compound exhibited stable protein–ligand interactions, favorable binding free energy, acceptable predicted pharmacokinetic properties, and balanced electronic stability throughout the analyses. Although the findings are computational and require experimental validation, CID-87014 emerged as a promising lead compound targeting AURKA and may serve as a foundation for future ovarian cancer drug development.

## Graphical abstract

**Figure:**
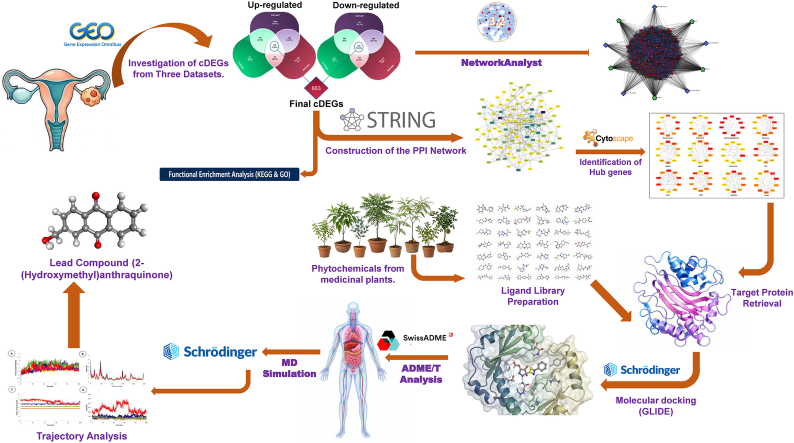

## 1 Background

Ovarian cancer (OC) remains one of the most lethal gynecological malignancies worldwide and continues to impose a major burden on women's health. Despite being less frequently diagnosed than breast or cervical cancer, OC is responsible for a disproportionately high number of cancer-related deaths among women because of its aggressive nature, frequent recurrence, and poor prognosis.^1^, ^2^ Approximately 90% of ovarian malignancies originate from the epithelial lining of the ovary.^3^ The poor survival outcome of OC is largely attributed to the absence of reliable early diagnostic biomarkers and the nonspecific clinical manifestations observed during the initial stages of disease progression.^4^ Consequently, most patients are diagnosed at advanced metastatic stages, where the five-year survival rate declines dramatically.^5^, ^6^ Although platinum-based chemotherapy combined with cytoreductive surgery remains the standard therapeutic strategy, the majority of patients eventually develop recurrence and chemoresistance, significantly limiting long-term treatment efficacy.^7^, ^8^, ^9^ Standard treatment primarily relies on cytoreductive surgery followed by platinum- and taxane-based chemotherapy, including drugs such as Cisplatin, Carboplatin, and Paclitaxel. Although these agents initially show favorable responses, many patients eventually develop chemoresistance, resulting in frequent relapse and poor long-term survival outcomes.^10^ Furthermore, conventional synthetic anticancer drugs are commonly associated with severe systemic toxicities, including nephrotoxicity, neurotoxicity, myelosuppression, and gastrointestinal complications, which significantly compromise patient tolerance and therapeutic efficacy.^11^ Therefore, the identification of novel molecular targets and safer therapeutic agents remains a critical priority in ovarian cancer research.^12^

Recent advances in high-throughput transcriptomic technologies, including microarray and RNA-sequencing platforms, have significantly improved our understanding of ovarian cancer biology. These technologies enable comprehensive identification of differentially expressed genes (DEGs) associated with tumor initiation, progression, metastasis, and therapeutic resistance.^13^ Previous transcriptomic investigations have demonstrated that dysregulation of genes involved in cell cycle progression, DNA replication, apoptosis, angiogenesis, immune signaling, and metastatic pathways contributes substantially to OC pathogenesis and disease progression.^14^, ^15^

However, interpreting gene-level data in isolation provides only a limited view of tumor biology.^16^ A more holistic approach involves systems biology, which considers how genes and their products interact within broader molecular networks.^17^ Protein–protein interaction (PPI) analysis helps identify central hub genes that hold regulatory control over multiple pathways, and network centrality metrics can pinpoint potential drug targets.^18^ Moreover, mapping the regulatory landscape using transcription factors (TFs) and microRNAs (miRNAs) can further elucidate the post-transcriptional and epigenetic regulation of DEGs.^19^

Natural products have long served as an important source of anticancer therapeutics because of their remarkable structural diversity and biological activities.^20^, ^21^ More than half of currently approved anticancer drugs are either natural products or their derivatives, including clinically important compounds such as paclitaxel, vincristine, and camptothecin.^22^, ^23^, ^24^ Phytochemicals are particularly attractive candidates because they often exhibit multitarget activities, lower toxicity profiles, and the ability to modulate diverse signaling pathways associated with proliferation, apoptosis, oxidative stress, inflammation, and metastasis.^25^ Several naturally occurring compounds, including curcumin, quercetin, resveratrol, and epigallocatechin gallate, have demonstrated promising anticancer activities against ovarian cancer in experimental studies.^26^, ^27^, ^28^ However, the precise molecular targets and pharmacological interactions of many phytochemicals remain insufficiently explored. The Indian Medicinal Plants, Phytochemistry and Therapeutics (IMPATT) database provides a valuable resource for exploring phytochemicals derived from medicinal plants and supports computational drug discovery investigations.^29^

Another major challenge in ovarian cancer treatment is the limited availability of target-specific therapeutic agents capable of overcoming tumor heterogeneity and chemoresistance. Recent studies suggest that integrating transcriptomic analyses with computational drug discovery strategies may accelerate the identification of clinically relevant molecular targets and candidate therapeutic compounds.^30^, ^31^, ^32^ Moreover, pan-cancer analyses have emerged as valuable approaches for evaluating whether dysregulated genes exhibit broad oncogenic relevance across multiple tumor types or possess cancer-specific significance.^33^ Such analyses improve biological interpretation and may enhance the translational potential of identified biomarkers and therapeutic targets.

**Fig. 1:**
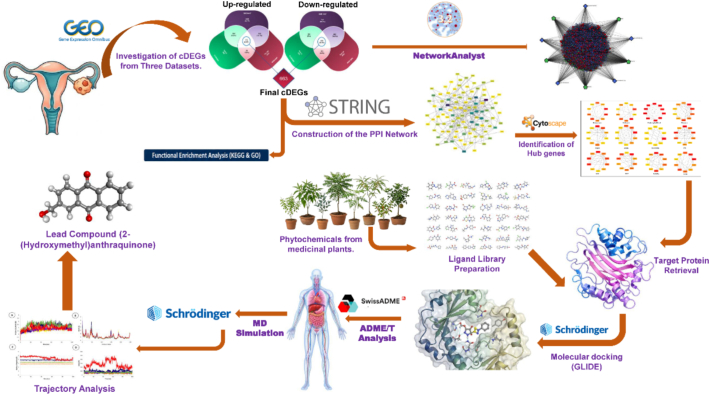
A detailed graphical representation depicting the conceptual framework, analytical workflow, and major outcomes of the study.

Therefore, the present study employed an integrative systems biology and computer-aided drug discovery approach to identify potential therapeutic targets and phytochemical inhibitors against ovarian cancer. Differential gene expression analysis was performed to identify significantly dysregulated genes, followed by functional enrichment and protein–protein interaction network analyses to determine key hub genes. Regulatory interactions involving TFs and miRNAs were further investigated to understand the underlying molecular control mechanisms. In addition, pan-cancer analyses were conducted to evaluate the broader oncogenic relevance and expression profiles of the identified targets. Subsequently, selected phytochemicals from the IMPATT database were screened against the top-ranked target using molecular docking, followed by ADMET, toxicity, and molecular dynamics simulation analyses to evaluate pharmacological stability and therapeutic potential. Through this integrative computational framework, the study aims to identify biologically relevant targets and promising phytochemical candidates that may contribute to the development of safer and more effective therapeutic strategies for ovarian cancer. The study design was graphically represented in (Fig. 1).

## 2 Materials and methods

### 2.1 Retrieval of microarray expression datasets

The microarray gene expression datasets used in this study were retrieved from the publicly available Gene Expression Omnibus (GEO) database, maintained by the National Center for Biotechnology Information (NCBI) (https://www.ncbi.nlm.nih.gov/geo/). Specifically, three OC-related datasets, GSE14407, GSE18520, and GSE26712 were selected for analysis. The GSE14407 dataset, based on the GPL570 platform (Affymetrix Human Genome U133 Plus 2.0 Array), contains a total of 24 samples, including 12 ovarian serous papillary carcinoma tissues and 12 normal ovarian surface epithelial samples.^34^ The GSE18520 dataset, also generated using the GPL570 platform, comprises 63 samples, including 53 advanced OC tissues and 10 normal controls.^35^ In addition, the GSE26712 dataset is based on the GPL96 platform (Affymetrix Human Genome U133A Array) and includes 195 samples, consisting of 185 primary OC tissues and 10 normal ovarian tissues.^36^, ^37^ These datasets were selected due to their relatively large sample sizes and availability of both tumor and normal tissue samples, enabling comprehensive comparative analysis.

### 2.2 Analysis of differentially expressed genes using GEO2R

To identify differentially expressed genes (DEGs) associated with OC, the NCBI GEO2R web tool (https://www.ncbi.nlm.nih.gov/geo/geo2r/) was utilized. Within each dataset, samples were categorized into two groups: ovarian cancer tissues (case) and normal ovarian tissues (control). The expression data were preprocessed and normalized using log2 transformation to ensure comparability across samples. The expression data were preprocessed and normalized using log2 transformation and force normalization (quantile normalization) in GEO2R to minimize technical variation and ensure comparability across samples. Differential expression analysis was performed using the Linear Models for Microarray Data (LIMMA) approach, which applies an empirical Bayes method to estimate gene-wise variability and improve statistical power.^38^ Genes with an adjusted *p*-value <0.05 and an absolute log fold change (|logFC|) > 1.0 were considered statistically significant DEGs. Genes with positive logFC values were regarded as upregulated, whereas those with negative logFC values were considered downregulated. Finally, the DEG lists obtained from the three datasets were compared to identify common differentially expressed genes (cDEGs). The overlap among these DEG sets was determined using the dplyr package in R^39^ and the common genes were visualized using Venn diagram. These steps enabled the identification of robust and consistently dysregulated genes across multiple datasets, thereby reducing dataset-specific bias. The resulting cDEGs are more likely to represent key molecular drivers of OC and serve as reliable candidates for further functional and therapeutic investigation.

### 2.3 Functional and pathway enrichment analyses of cDEGs

The Gene Ontology (GO) and KEGG pathway enrichment analyses were performed using the DAVID database^40^ to explore the biological significance of the cDEGs. GO terms classified the genes based on molecular functions, biological processes, and cellular components, while KEGG identified relevant signaling pathways. The threshold for GO terms was set at a minimum gene count ≥2 and *p*-value <0.05. Enriched KEGG pathways were selected based on DAVID's default EASE score to highlight significant functional categories. These enrichment analyses provide critical insights into the underlying biological mechanisms associated with OC by translating large gene lists into functionally interpretable categories. Specifically, GO analysis helps to identify key biological processes, molecular activities, and cellular localizations that are disrupted in cancer, while KEGG pathway analysis reveals the major signaling pathways potentially driving tumor initiation and progression. This integrative functional interpretation not only enhances the understanding of disease pathogenesis but also aids in identifying biologically relevant pathways and potential molecular targets for therapeutic intervention.

### 2.4 Regulatory analysis of cDEGs at transcriptional and posttranscriptional levels

We performed a combined regulatory network analysis to identify both transcriptional and post-transcriptional regulators of the cDEGs. Transcription factors (TFs) were predicted using TF–target interactions from the JASPAR database,^41^ while miRNA–target interactions were retrieved from miRTarBase.^42^ Key regulators were identified based on their topological properties within the network. This integrative network analysis provides insight into the upstream regulatory mechanisms controlling gene expression alterations in OC. Identification of key TFs and miRNAs highlights potential master regulators that may play critical roles in disease progression and could serve as promising targets for future therapeutic interventions.

### 2.5 PPI network analysis for key hub genes discovery

After identifying the common differentially expressed genes (cDEGs), we explored the interactions among their encoded proteins using the STRING database.^43^ A full STRING network was selected, where edges represent both functional and physical protein associations. The interaction confidence was defined based on confidence scores, with a minimum required interaction score set to medium confidence (0.400). To ensure the reliability of the interactions, only experimentally validated evidence sources were included, while other sources such as text mining, co-expression, and database predictions were excluded. Additionally, to focus strictly on the interactions among the input genes, no additional interactors were added (the network was limited to query proteins only). The network was then visualized in Cytoscape (v3.10.4), where each node represents a protein and each edge represents an interaction.^44^ To identify the most important genes within the network, the CytoHubba plugin was used. Twelve topological algorithms, such as Degree, MCC, MNC, DMNC, EPC, ClusteringCoefficient, Radiality, Stress, Betweenness, Bottleneck, Eccentricity, and Closeness, were applied to rank the genes based on their network characteristics.^45^. Rather than relying on a single method, we selected hub genes based on their consistent appearance across multiple topological approaches. In particular, genes that were commonly identified by most of the methods were considered as key hub genes. This approach improves the reliability of the results and helps highlight genes that are more likely to play central roles in OC progression.

### 2.6 Pan-cancer analysis

#### 2.6.1 Transcriptional expression analysis of the top hub gene in ovarian and other cancers

The transcriptional expression profile of the top-ranked hub gene was evaluated across OC and other tumor types using the Gene_DE module of TIMER2.0 database^46^ (http://timer.comp-genomics.org/). For cancers lacking matched normal tissues in TCGA, additional expression comparisons were obtained from GEPIA2 database^47^ (http://gepia2.cancer-pku.cn/#analysis), which integrates TCGA and GTEx datasets. Expression differences between tumor and normal tissues were analyzed using ANOVA, with values log2 (TPM + 1) transformed. For expression analysis using GEPIA2, the following parameters were applied: Log2FC cutoff = 1, *p*-value cutoff = 0.01, and jitter size = 0.4. It should be highlighted that TIMER2.0 and GEPIA2 are built upon different data resources. TIMER2.0 is based solely on reprocessed TCGA RNA-seq data,^46^ while GEPIA2 integrates both TCGA and GTEx datasets, harmonized under a single processing pipeline.^47^ Hence, slight inconsistencies in expression results across cancer types may arise between the two platforms.

#### 2.6.2 Protein expression analysis of the top hub gene

The protein expression pattern of the top-ranked hub gene was examined across multiple tumor types, including OC, using the CPTAC dataset available in the UALCAN portal (http://ualcan.path.uab.edu/analysisprot.html).^48^ This resource integrates Clinical Proteomic Tumor Analysis Consortium (CPTAC) data to provide differential protein expression between tumor and normal tissues. Comparative analyses were performed to evaluate significant alterations in protein abundance of the hub gene across cancers.

#### 2.6.3 Correlation analysis of the top hub gene with other hub genes

To investigate the association between the most significant hub gene and the other top hub genes, a correlation analysis was performed using the Gene_Corr module of the TIMER2.0 web server across TCGA pan-cancer datasets. Specifically, the top-ranked hub gene was analyzed for its correlation with the remaining nine hub genes within the top 10 list. The resulting heatmap illustrates the purity-adjusted partial Spearman's rho values, reflecting the strength and direction of their correlations. The “Purity Adjustment” option was applied to minimize the confounding effect of tumor purity, thereby providing unbiased correlation estimates across diverse cancer types.

#### 2.6.4 Immune infiltration analysis of the top hub gene

The “Immune-Gene” module of TIMER2.0 was employed to explore the relationship between the top-ranked hub gene and immune cell infiltration across TCGA cancers. In this analysis, particular attention was given to CD4+ T cells, macrophages, and mast cells. The estimation of immune infiltration was derived from multiple deconvolution algorithms, while purity-adjusted Spearman's correlation provided both *p*-values and correlation coefficients. Results were visualized as heatmaps to illustrate the associations.

### 2.7 Preparation of the top-ranked hub protein

Following PPI network analysis, the top-ranked hub protein was selected for structural preparation. Its crystallographic structure was retrieved from the RCSB PDB database.^49^ The retrieved protein structure was prepared following a standard refinement protocol in Schrödinger's Protein Preparation Wizard (v12.5, Schrödinger 2020–3, LLC, New York, NY). Bond orders were assigned using the CCD database, and hydrogen atoms were added. Metal zero-order bonds and disulfide linkages were defined, while missing loops and side chains were rebuilt with Prime. The termini were capped, and water molecules located more than 5 Å from the active site were removed. Protonation states were generated at pH 7.0 ± 2.0 using Epik. Hydrogen-bond networks were optimized with PROPKA at pH 7.0, and the OPLS3e force field was applied.^50^ Energy minimization was carried out with heavy-atom convergence constrained to an RMAD of 0.30 Å.

### 2.8 Retrieval and preparation of ligand compounds

To explore possible inhibitors, 6834 phytochemicals obtained from 25 plant species native to the Indian subcontinent were analyzed in this research (**Table S1**). These plants, commonly used in traditional medicine across the Indian subcontinent, were selected based on literature support and availability in the IMPPAT database (https://cb.imsc.res.in/imppat/). The compounds were downloaded in SDF format. Prior to screening, the raw compound library was curated by removing duplicate entries and redundant structures, yielding unique phytochemicals for downstream analysis. The ligands were prepared using the LigPrep application,^51^ applying minimization through the OPLS3e force field. Both Epik and the ionizer were employed to generate possible states within a standard pH range of 7.0 ± 2.0. For each structure, up to 32 conformers were generated, maintaining an RMSD cutoff of 1.0 Å. In this study, Y3M was selected as the control ligand because it is an experimentally validated inhibitor of Aurora A kinase (PDB ID: 4UYN), providing a reliable structural and interaction benchmark for evaluating the binding affinity and pose of newly screened phytochemical compounds.

### 2.9 Assessment of binding affinity via molecular docking

We conducted site-specific molecular docking with Maestro (Schrödinger Release 2021–2, Schrödinger LLC, New York, NY, USA, 2023), applying the extra precision (XP) mode. After docking, the PDB files for each protein-ligand complex were exported from the Maestro trajectory outputs and used for post-docking analysis.^52^, ^53^ The post-docking workflow assessed non-covalent contacts, hydrophobic features, and qualitative bioactivity. Hydrogen bonds and hydrophobic interactions were mapped with LigPlot+ version 2.2 running on Java SE Runtime Environment 8u271, which accepts concatenated PDB files produced by Maestro. Binding compactness, the distribution of polar and non-polar contacts, and overall complex geometry were examined in Discovery Studio Visualizer 64-bit (http://media.accelrys.com/downloads/visualizer/45/DS45Client.exe) to confirm the interaction patterns.

### 2.10 Evaluation of drug-likeness, ADMET, and toxicity

Pharmacokinetic properties are essential for determining the drug-likeness and early-stage suitability of candidate compounds in Computer-Aided Drug Design (CADD).^54^ In this study, the top 20 phytochemicals with the highest docking scores were selected for ADMET analysis. The SwissADME (http://www.swissadme.ch/) server was utilized to examine crucial pharmacokinetic features, including physicochemical characteristics, lipophilicity, water solubility, gastrointestinal (GI) absorption, and drug-likeness.^55^ To assess toxicological risks, the pkCSM server (https://biosig.lab.uq.edu.au/pkcsm/) was employed. This tool predicted various toxicity endpoints such as hepatotoxicity, mutagenicity, cardiotoxicity, and skin toxicity, enabling the identification of compounds with comparatively favorable predicted toxicity profiles for further evaluation.^56^

### 2.11 Molecular dynamics simulation

Molecular dynamics involves simulating the motion and interactions of protein–ligand complexes under conditions that mimic a physiological environment.^57^ To examine the thermodynamic compatibility and stability of these protein–ligand complexes, 150 ns molecular dynamics (MD) simulations were carried out using the paid version of Schrödinger's Desmond v3.6 program within a Linux environment. System preparation involved the predefined TIP3P water model within an orthorhombic periodic boundary box with a 10 Å buffer distance. Appropriate counter-ions and 0.15 M NaCl were added to neutralize the system and replicate physiological conditions. The solvated systems were first energy-minimized using the steepest descent algorithm, followed by a two-step equilibration process consisting of NVT and NPT ensembles to stabilize temperature and pressure using the OPLS3e force field parameters in Desmond. The NPT ensemble was then used for the production run, maintaining constant conditions of 300 K and 1.01325 bar, with temperature and pressure controlled using the Nose–Hoover thermostat and Martyna–Tobias–Klein barostat. Trajectory frames were recorded every 100 ps throughout the 150 ns simulation.^33^

#### 2.11.1 Analysis of simulation trajectories

Molecular dynamics simulation images were generated using the Schrödinger Maestro application (version 9.5). To examine potential simulation scenarios and evaluate the reliability of the results, the Simulations Interaction Diagram (SID) from the Desmond module was applied. Parameters such as molecular surface area (MolSA), polar surface area (PSA), root-mean-square deviation (RMSD), protein–ligand contacts (P–L), root-mean-square fluctuation (RMSF), and radius of gyration (Rg) were analyzed to assess the stability and convergence of the protein–ligand complex throughout the simulation trajectory.

### 2.12 Post simulation MMGBSA

Binding free energies of the protein–ligand complexes throughout the 150 ns simulation were estimated using the thermal_mmgbsa.py module from Schrödinger's Prime package. Snapshots were extracted from the Desmond molecular dynamics trajectory, and MM-GBSA calculations were carried out for each snapshot. The protocol internally separates the complex into receptor and ligand components to compute the associated energy terms. A total of 20 snapshots were extracted from the final stage of the trajectory at regular intervals, and the average binding free energy was calculated.

### 2.13 Quantum mechanical (QM) analysis

Quantum mechanical (QM) calculations were performed using Jaguar v10.9 with density functional theory (DFT)^58^ to analyze the electronic properties of selected ligands. The B3LYP functional^59^ combined with the Lee–Yang–Parr correlation^60^ and the 6-31G(d,p) basis set was applied to optimize ligand geometries and evaluate frontier molecular orbitals (HOMO and LUMO). These orbital energies were used to determine ionization potential, electron affinity, energy gap, chemical hardness, chemical potential, and softness following Koopmans' theorem and the Parr–Pearson approach^61^, ^62^ A lower hardness value indicates higher reactivity, whereas greater softness reflects enhanced electron-accepting capability. These descriptors provide insight into the stability, reactivity, and electron transfer potential of ligand–protein complexes.^63^

$$E_{g}=E_{\text{LUMO}}-E_{\text{HOMO}}$$

$$I=\;--E_{\text{HOMO}}$$

$$A=\;--E_{\text{LUMO}}$$

$$\eta \;=\frac{1}{2}\left(I-A\right)$$

$$S\;=\frac{1}{\eta }$$

To evaluate the electronic behavior of the ligands, key quantum descriptors such as energy gap (E_g_), ionization potential (I), electron affinity (A), global hardness (η), and global softness (S) were calculated. These parameters help clarify the stability, reactivity, and electron-donating or electron-accepting tendencies of the compounds.

## 3 Result

### 3.1 Detection and selection of DEGs

**Fig. 2:**
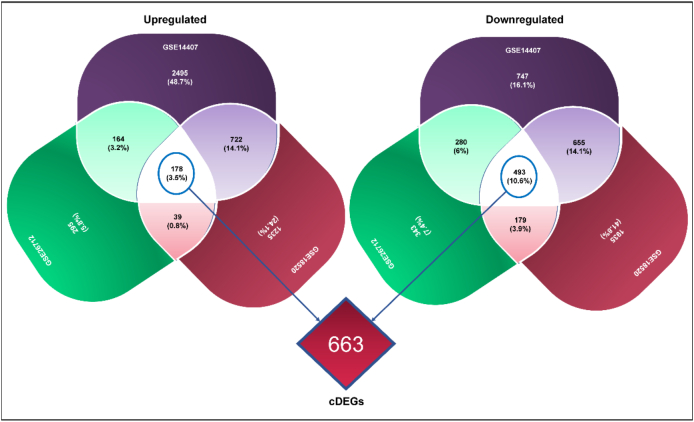
Graphical visualization illustrating the overlap of differentially expressed genes (DEGs) identified from three independent datasets.

Differential gene expression analysis was carried out for the three selected datasets: GSE14407, GSE18520, and GSE26712. Each dataset was analyzed separately to identify genes that were significantly upregulated or downregulated in OC samples compared to normal tissues. In the GSE14407 dataset, a total of 3568 genes were found to be upregulated, while 2179 genes were downregulated. For GSE18520, 2179 genes showed upregulation and 3274 genes were downregulated. In the case of GSE26712, 677 genes were identified as upregulated and 1297 genes were downregulated (**Table S2**). The overlap of DEGs among the three datasets is illustrated in Fig. 2 using a Venn diagram. The diagram clearly shows the distribution of both unique and shared genes across GSE14407, GSE18520, and GSE26712. From this analysis, 178 commonly upregulated genes and 493 commonly downregulated genes were identified across all three datasets (**Table S3**). The consistent dysregulation of these genes across datasets strengthens their biological relevance and minimizes dataset-specific bias. These cDEGs likely represent core molecular alterations associated with OC pathogenesis and therefore provide a robust foundation for downstream functional, network, and target-identification analyses.

### 3.2 Functional and pathway enrichment analyses of DEGs

Functional enrichment analysis demonstrated that the identified cDEGs were predominantly associated with cell-cycle regulation, mitotic progression, chromosome segregation, and cellular proliferation. These biological processes are well-recognized hallmarks of ovarian cancer progression and support the notion that uncontrolled cell division is a central feature of the disease. KEGG pathway analysis further highlighted significant enrichment in pathways related to cell cycle control, p53 signaling, DNA repair, and PI3K-Akt signaling, suggesting that the dysregulated genes participate in interconnected oncogenic networks that promote tumor growth, survival, and genomic instability. The complete list of significantly enriched terms for the cDEGs is provided in **Table S4**.

### 3.3 Identification of regulatory biomolecules

**Fig. 3:**
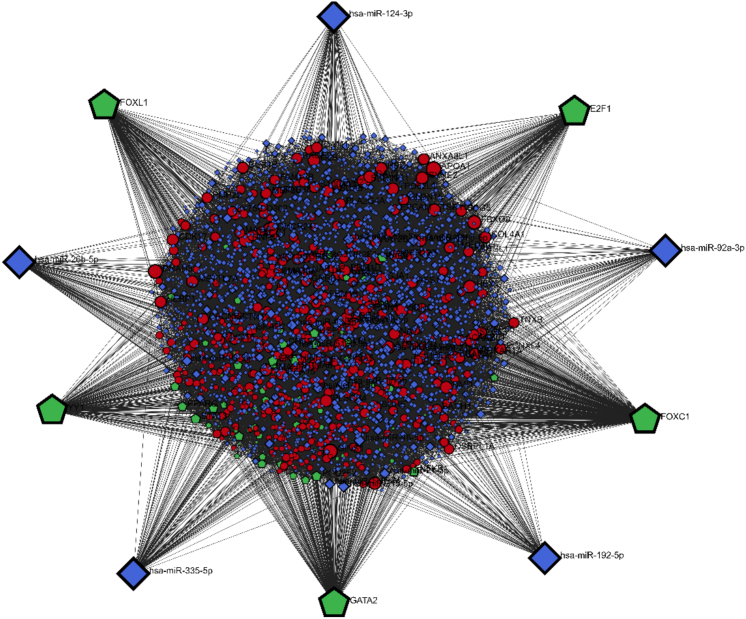
Integrated regulatory network of cDEGs, transcription factors (TFs), and microRNAs (miRNAs) in ovarian cancer. Red circles represent DEGs, green diamonds denote TFs, and blue pentagons indicate miRNAs. (For interpretation of the references to colour in this figure legend, the reader is referred to the web version of this article.)

To investigate the regulatory mechanisms of the identified cDEGs, we performed an integrated analysis of their interactions with transcription factors (TFs) and microRNAs (miRNAs). The combined interaction network is presented in Fig. 3, where both DEG–TF and DEG–miRNA interactions were visualized. Network analysis identified FOXC1, GATA2, YY1, FOXL1, and E2F1 as the most influential transcriptional regulators, while hsa-miR-335-5p, hsa-miR-26b-5p, hsa-miR-124-3p, hsa-miR-192-5p, and hsa-miR-92a-3p emerged as major post-transcriptional regulators. Several of these regulatory molecules have previously been implicated in cancer progression, cell-cycle control, and metastatic behavior. Their central positions within the regulatory network suggest that they may coordinate the expression of multiple ovarian cancer–associated genes and contribute to disease development through both transcriptional and post-transcriptional mechanisms.

### 3.4 Analysis of PPI network

**Fig. 4:**
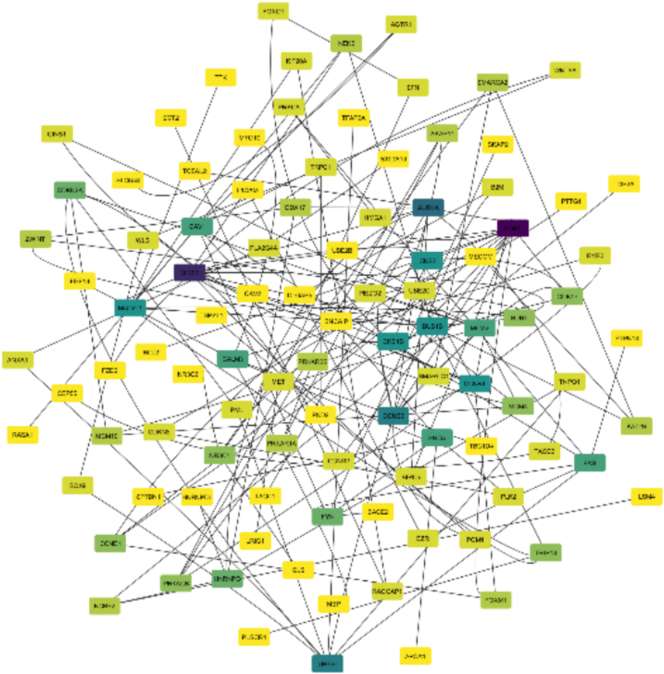
Construction of the protein–protein interaction (PPI) network of common differentially expressed genes (cDEGs) using the STRING database and visualization in Cytoscape.

**Fig. 5:**
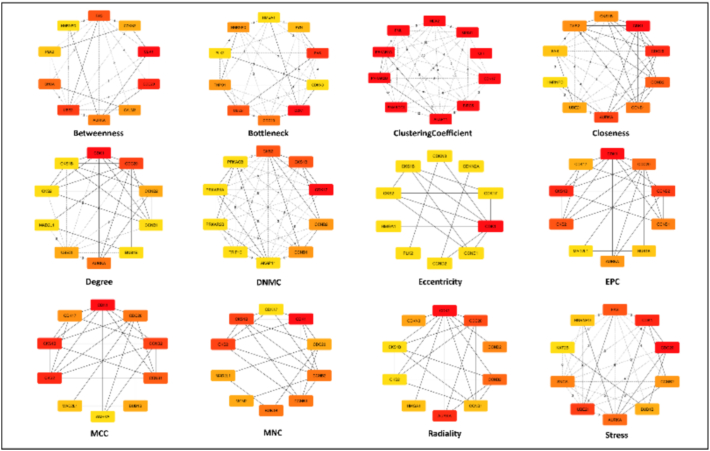
Hub genes identified by twelve CytoHubba algorithms based on topological analysis of the PPI network.

The overall interaction network of the common DEGs is shown in Fig. 4. In this network, disconnected nodes were excluded to improve network clarity where nodes represent proteins and edges indicate their interactions. The network reveals a strong level of connectivity among the identified genes. To identify the most important genes, topological analysis was performed using the CytoHubba plugin. Twelve different algorithms were applied to rank the genes. The top 10 genes obtained from each method are presented in Fig. 5. After comparing the results from all methods, several genes appeared repeatedly. Among them, CDK1, CDC20 and AURKA were the most frequently identified genes across multiple algorithms. This repeated occurrence suggests that these genes have central roles in the PPI network and may be crucial in OC progression. Although CDK1, CDC20, and AURKA are all involved in cell cycle regulation and exhibited high network centrality, CDK1 was deprioritized because of its essential role in normal cell cycle progression, where it regulates multiple critical processes including mitotic entry, chromosome segregation, and genome stability.^64^ CDC20 was also considered less suitable due to its primary function as a regulatory cofactor of the anaphase-promoting complex/cyclosome (APC/C), mediating protein–protein interactions that control mitotic progression.^65^ Such interaction-driven targets are traditionally considered challenging targets for small-molecule drug design due to the lack of well-defined binding pockets. In contrast, AURKA, a serine/threonine kinase involved in mitotic regulation, is frequently overexpressed in various cancers and is associated with chromosomal instability and tumor progression.^65^ Moreover, AURKA contains a structurally well-defined kinase domain, and several small-molecule inhibitors targeting this domain have already been developed, supporting its druggability and translational potential. Therefore, AURKA was selected as the most suitable and therapeutically relevant target among the candidates.

### 3.5 Pan-cancer analysis

#### 3.5.1 Transcriptional and protein expression analysis of the top hub gene

**Fig. 6:**
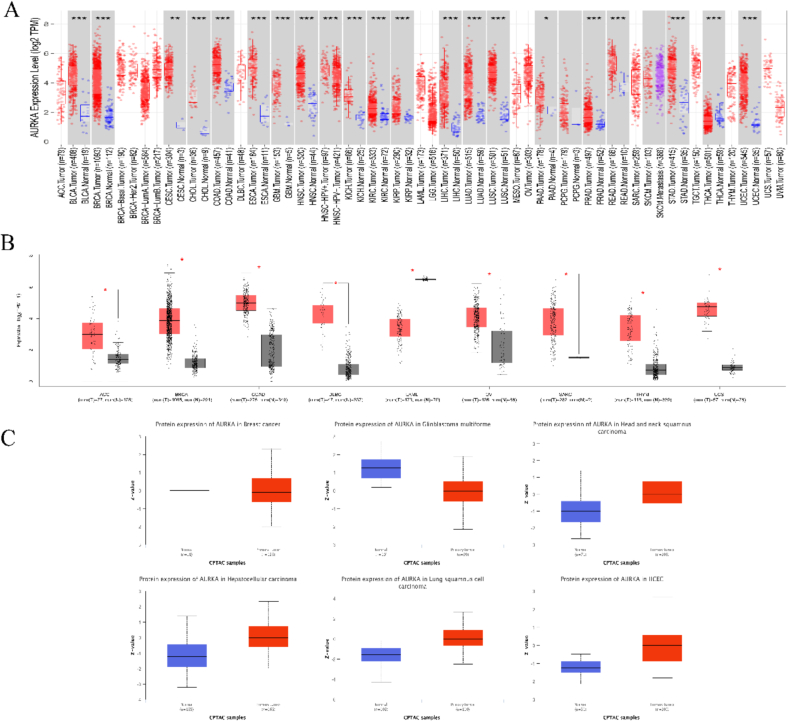
Transcriptional and proteomic expression analysis of AURKA across human cancers (A) TIMER2.0 analysis showing AURKA mRNA expression levels across various cancer types, where significance levels are indicated as ***P < 0.001, **P < 0.01, and *P < 0.05. (B) GEPIA2 validation identifying additional tumor types with significant AURKA expression differences not covered by TIMER2.0. (C) UALCAN proteomic data showing significantly elevated AURKA protein expression in multiple cancers compared with normal tissues.

The transcriptional expression profiling of AURKA across different cancer types was analyzed using the TIMER2.0 database, as illustrated in Fig. 6A. In the figure, three stars (***) indicate highly significant overexpression (*P* < 0.001), two stars (**) represent moderate significance (*P* < 0.01), while a single star (*) denotes relatively lower but statistically significant expression differences (*P* < 0.05). To further validate, GEPIA2 analysis revealed nine additional tumor types, not covered by TIMER2.0, that showed significant AURKA expression differences (Fig. 6B). Although TIMER2.0 and GEPIA2 largely agreed in showing AURKA overexpression across various tumor types, slight discrepancies were noted in some cancers, likely due to differences in their underlying datasets (TCGA-only for TIMER2.0 vs. TCGA+GTEx for GEPIA2). Additionally, to evaluate AURKA at the protein level, its proteomic profiles were analyzed using the UALCAN database. The results showed significantly higher AURKA protein expression in multiple tumor types compared with normal tissues (Fig. 6C).

#### 3.5.2 Correlation analysis of the top hub gene with other hub genes

**Fig. 7:**
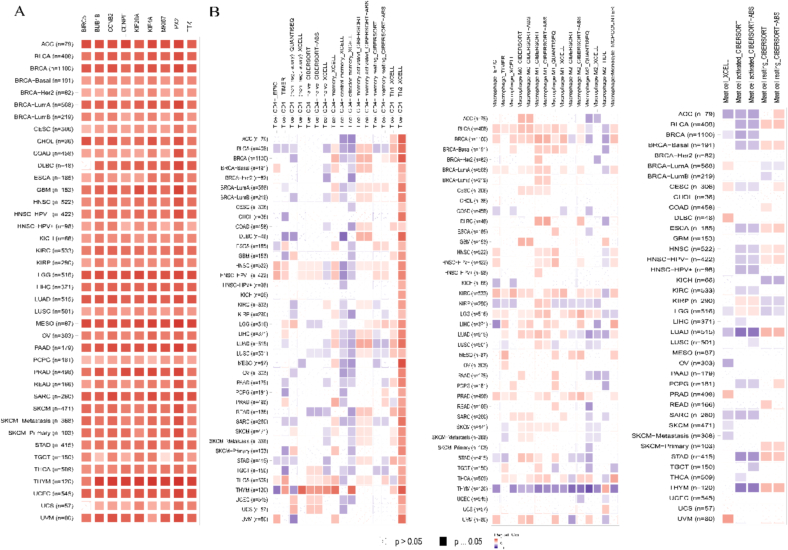
Correlation and immune infiltration analyses of AURKA using TIMER2.0. (A) Heatmap illustrating tumor purity–adjusted partial Spearman's correlation coefficients (ρ) between AURKA and other top-ranked hub genes across TCGA cancer types. (B) Heatmaps depicting tumor purity–adjusted Spearman's correlations between AURKA expression and the infiltration levels of CD4^+^ T cells, macrophages, and mast cells across TCGA cancer types.

Correlation analysis using the TIMER2.0 Gene_Corr module demonstrated that AURKA showed consistently strong positive correlations with all other top hub genes. The purity-adjusted partial Spearman's rho values confirmed that these associations were robust across diverse TCGA cancer types, minimizing the confounding impact of tumor purity. These results suggest that AURKA is centrally positioned within the hub gene network and may coordinate shared oncogenic pathways. The complete correlation profile is illustrated in Fig. 7A as a heatmap. The widespread positive correlations observed between AURKA and other hub genes suggest the presence of coordinated regulatory programs involved in cell-cycle progression and mitotic regulation. This central positioning further reinforces the biological significance of AURKA within the ovarian cancer molecular network.

#### 3.5.3 Immune infiltration analysis of the top hub gene

Immune infiltration analysis revealed context-dependent associations between AURKA expression and immune cell populations across TCGA cancer types. Negative correlations between AURKA and mast cell infiltration were predominantly observed in several epithelial cancers, including BRCA subtypes, KICH/KIRP, LUAD, and STAD, particularly in activated mast cell estimations generated by the CIBERSORT and CIBERSORT-ABS algorithms. In contrast, positive correlations were detected in certain tumor types such as UVM, LUAD, and STAD, mainly in resting mast cell analyses using the CIBERSORT and XCELL methods. These findings suggest that the relationship between AURKA expression and mast cell infiltration may vary depending on the tumor microenvironment and cancer context. Similarly, macrophage infiltration analysis demonstrated positive correlations between AURKA expression and macrophage populations in several cancers. The strongest associations were observed for Macrophage_M0_CIBERSORT in GBM and Macrophage_M1_CIBERSORT in BRCA, while moderate positive correlations were identified in PAAD, UVM, and KIRC using the EPIC algorithm. In contrast, THYM exhibited predominantly negative correlations across most estimation methods, indicating distinct immune characteristics in this cancer type. Among the evaluated immune infiltration methods, the Th2_XCELL algorithm consistently demonstrated positive correlations between AURKA expression and CD4+ T-cell infiltration across multiple cancer types (*p* < 0.05). Overall, these findings indicate that AURKA expression is associated with diverse immune infiltration patterns in a cancer-specific manner, highlighting its potential relevance in tumor–immune interactions (Fig. 7B).

### 3.6 Structural analysis of the top-ranked hub protein

The crystallographic structure of AURKA (PDB ID: 4UYN) was retrieved from the RCSB Protein Data Bank^66^ and subsequently refined as described in the methodology section, including heteroatom and water removal and energy minimization for downstream applications.

### 3.7 Assessment of binding affinity via molecular docking

From the original collection of 6834 phytochemicals, removal of duplicate structures resulted in a refined set of 2046 unique ligands used for docking. These were screened against 4UYN using molecular docking to identify potential inhibitors, using a grid box centered at the binding site with coordinates X = 26.746, Y = −5.062, and Z = 14.877. To validate the docking protocol, redocking of the co-crystallized ligand (Y3M) was performed, yielding a low heavy atom RMSD value of 0.43 Å, which confirmed the reliability of the docking setup in reproducing the native binding pose (Fig. S1). Based on the binding affinity scores, the top 10 phytochemicals and control were shortlisted, exhibiting docking scores ranging from −8.155 to −7.634 kcal/mol. The control compound (Y3M) exhibited the strongest docking affinity (−8.449 kcal/mol); however, several phytochemicals showed comparable binding poses and were therefore advanced for multi-parameter evaluation. These selected compounds, along with the control and their docking scores were presented in Table 1, while the complete docking results of all screened compounds were provided in **Table S5**.

**Table 1:** Top 10 phytochemicals with the highest binding affinities against 4UYN, ranked by docking score (kcal/mol).

IMPPAT IDs	PubChem CIDs	Phytochemical Names	Source	2D Structures	Binding Affinity (Maestro) (kcal/mol)	Binding Affinity (Autodock Vina) (kcal/mol)
IMPHY014243	12,310,964	Rhababerone	*Aloe vera*		−8.155	−8.3
IMPHY006310	6037	Folic	*Allium sativum*		−8.103	−8.4
IMPHY011735	65,084	(+)-Gallocatechin	*Camellia sinensis*		−8.089	−7.2
IMPHY001944	11,597,485	Glychionide A	*Glycyrrhiza glabra*		−8.007	−9
IMPHY010266	11,699,925	(+)-Catechin-(4alpha- > 8)-(−)-epigallocatechin	*Camellia sinensis*		−8.003	−8
IMPHY014966	5,282,149	Trifolin	*Panax ginseng*		−7.969	−7.5
IMPHY011543	3220	Emodin	*Aloe vera*		−7.820	−8
IMPHY004857	442,877	Withasomnine	*Withania somnifera*		−7.750	−6.5
IMPHY006933	87,014	2-(Hydroxymethyl)anthraquinone	*Curcuma longa*		−7.672	−8.3
IMPHY011985	5,318,767	Nicotiflorin	*Azadirachta indica*		−7.634	−8.3
Y3M (Control)	86,278,048	Ethyl (9*S*)-9-[5-(1H-benzimidazol-2-ylsulfanyl)furan-2-yl]-8-oxo-3,4,5,6,7,9-hexahydropyrrolo[3,4-*b*]quinoline-3-carboxylate	Synthetic compound		−8.449	−8.8

To further validate the reliability and consistency of the docking results, the selected top-ranked phytochemicals were additionally evaluated using an independent docking platform. Comparative analysis demonstrated that all shortlisted compounds retained significant binding affinity toward the target protein across both docking approaches, supporting the robustness and reproducibility of the predicted ligand–protein interactions. These findings strengthened the confidence in the selected compounds as potential inhibitors for subsequent analyses (Table 1).

### 3.8 Evaluation of drug-likeness, ADMET, and toxicity

The ADMET profiles of the ten docked compounds and control were provided in **Table S6**. From these, five compounds (CID-12310964, CID-65084, CID-3220, CID-442877, and CID-87014) were selected for further evaluation based on their pharmacokinetic properties. The assessed parameters included rotatable bonds, molecular weight (MW), gastrointestinal absorption, number of heavy atoms, hydrogen bond acceptors, and hydrogen bond donors, as presented in Table 2. Lipinski's Rule of Five was met by all selected compounds, and the majority of ADMET characteristics remained within the standard thresholds. Although the control compound showed strong docking affinity, its larger molecular weight, low GI absorption, and predicted toxicity liabilities made several phytochemicals comparatively more favorable for drug development. The toxicity profiles, including AMES mutagenicity, skin sensitization, hepatotoxicity, and hERG II inhibition (a predictor of cardiotoxicity), were compiled in Table 2.

**Table 2:** Comprehensive analysis of absorption, distribution, metabolism, excretion, and toxicity (ADMET) properties of the selected phytochemicals.

Properties		IMPHY014243 (CID-12310964)	IMPHY011735 (CID-65084)	IMPHY011543 (CID-3220)	IMPHY004857 (CID-442877)	IMPHY006933 (CID-87014)	Y3M (CID-86278048)
**Physico-chemical Properties**	**MW (g/mol)**	270.24	306.27	270.24	184.24	238.24	474.53
	**Consensus LogP**	2.01	0.52	1.87	2.37	2.22	3.04
	**H-bond acceptors**	5	7	5	1	3	6
	**H-bond donors**	3	6	3	0	1	2
	**Rotatable bonds**	0	1	0	1	1	6
	**Polar surface area**	94.83	130.61	94.83	17.82	54.37	134.88
**Pharmacokinetics**	**ESOL Log S**	−4.02	−2.08	−3.67	−2.9	−3.44	−4.72
	**GI absorption**	High	High	High	High	High	Low
	**BBB permeant**	No	No	No	Yes	Yes	No
	**CYP1A2 inhibitor**	Yes	No	Yes	Yes	Yes	No
	**CYP2C19 inhibitor**	No	No	No	No	No	Yes
	**CYP2C9 inhibitor**	No	No	No	No	No	Yes
	**CYP2D6 inhibitor**	No	No	No	No	No	No
	**CYP3A4 inhibitor**	Yes	No	Yes	No	No	Yes
**Drug likeness**	**Lipinski violations**	Accepted	Accepted	Accepted	Accepted	Accepted	Accepted
	**Bioavailability**	0.55	0.55	0.55	0.55	0.55	0.55
**Medi. Chemistry**	**Synth. accessibility**	2.53	3.53	2.57	2.2	2.34	5.13
**Toxicity**	**AMES toxicity**	Yes	Yes	No	Yes	Yes	Yes
	**Hepatotoxicity**	No	No	No	No	No	Yes
	**Skin Sensitisation**	No	No	No	No	No	No
	**hERG II inhibitor**	No	No	No	No	No	Yes

### 3.9 Quantum mechanical calculation analysis

The HOMO–LUMO distributions of the ligands were shown in **Fig. S2**, and the calculated quantum descriptors were summarized in Table 3. In quantum chemical terms, the HOMO–LUMO energy gap reflects molecular stability and ease of charge transfer, where smaller gaps generally indicate higher chemical reactivity, while larger gaps suggest greater stability.^67^ Hardness and softness further describe resistance or adaptability to electronic redistribution during intermolecular interactions.^67^ CID-87014 exhibited a relatively low energy gap (0.153 eV), moderate hardness (0.0768), and high softness (13.01), indicating a favorable balance between structural stability and electronic responsiveness. In a biological environment, such balanced behavior may support stable binding within the protein pocket while still allowing efficient charge-transfer interactions with surrounding amino acid residues, which can enhance binding affinity and complex stabilization.^68^ CID-442877 and CID-65084 showed wider gaps (0.200–0.210 eV) and higher hardness values, suggesting greater intrinsic stability but lower reactivity. Notably, CID-65084 exhibited the largest HOMO-LUMO gap and highest hardness among the evaluated compounds, indicating comparatively reduced electronic adaptability that may limit its interaction potential with the target protein.^67^ In contrast, CID-3220 and CID-12310964 displayed the smallest gaps (0.137 eV and 0.128 eV) with lower hardness, indicating higher reactivity that could favor interactions but may also correspond to reduced electronic stability under physiological conditions. Although CID-12310964 demonstrated favorable electronic reactivity, its overall suitability was limited by less favorable pharmacokinetic characteristics, particularly its predicted CYP450 inhibition profile. Compared with the control ligand (gap 0.142 eV; hardness 0.071), CID-87014 demonstrated a more balanced electronic profile, combining sufficient stability with favorable reactivity. Together with the favorable electronic characteristics of CID-442877 and the low predicted toxicity of CID-3220, these findings supported the selection of CID-87014, CID-442877, and CID-3220 for subsequent molecular dynamics simulations and binding free-energy analyses.

**Table 3:** Calculated quantum chemical descriptors of the ligands, including HOMO and LUMO energies, energy gap (Eg), ionization potential (I), electron affinity (A), global hardness (η), and softness (S).

**Molecules**	**εHOMO**	**εLUMO**	**Gap**	**Η (Hardness)**	**S (Softness)**
CID-442877	−0.207186	−0.007167	0.200019	0.1000095	9.99905009
CID-87014	−0.256743	−0.103106	0.153637	0.0768185	13.01769756
CID-3220	−0.228254	−0.090801	0.137453	0.0687265	14.55042815
CID-65084	−0.200542	0.010119	0.210661	0.1053305	9.493926261
CID-12310964	−0.218211	−0.089868	0.128343	0.0641715	15.58324178
CID-86278048 (Control)	−0.209442	−0.066651	0.142791	0.0713955	14.006485

### 3.10 Intermolecular interaction analysis

**Fig. 8:**
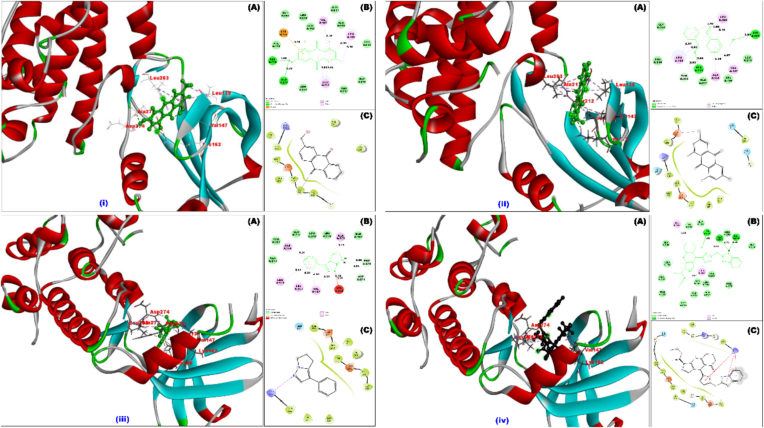
Three-dimensional (3D) and two-dimensional (2D) molecular docking interaction profiles of the top three candidate ligands and the reference ligand within the active-site binding pocket of the target protein. Panels correspond to (i) CID-3220, (ii) CID-442877, (iii) CID-87014, and (iv) the reference ligand Y3M. For each ligand, panel (A) depicts the 3D binding pose within the active site, panel (B) presents the detailed 2D interaction map (BIOVIA Discovery) highlighting key amino acid residues and bond distances (Å), and panel (C) illustrates a simplified 2D representation (Maestro) of the ligand-binding microenvironment.

Based on the overall evaluation of docking performance, ADMET properties, and quantum profiles, CID-3220, CID-442877, and CID-87014 were selected for further analyses. Interaction analysis (Fig. 8**)** demonstrated that the candidate ligands occupied a common binding pocket characterized by recurrent contacts with LEU263, VAL147, LEU139, ALA273, ASP274, and LYS162. CID-3220 formed hydrogen bonds with ASP274 and ALA273 and hydrophobic contacts with LEU263, VAL147, and LEU139. CID-442877 established hydrogen bonds with LYS162, TYR212, and ALA213, together with hydrophobic interactions involving LEU263, LEU139, VAL147, and ALA160. CID-87014 formed hydrogen bonds with ASP274 and PHE275 and exhibited extensive hydrophobic interactions with ALA160, ALA273, LEU139, VAL147, and LEU263. Although a single unfavorable interaction with LYS162 was observed, this interaction is unlikely to substantially compromise binding because it was accompanied by multiple stabilizing hydrophobic contacts and key hydrogen bonds within the active site. The extensive interaction network of CID-87014 may therefore contribute to its favorable binding orientation and overall complex stability. The reference ligand Y3M formed hydrogen bonds with LYS162, ALA273, and ASP274 and hydrophobic interactions with LEU263 and VAL147. Collectively, the repeated involvement of LEU263 and VAL147 in hydrophobic stabilization and ALA273 and ASP274 in hydrogen bonding suggests that these residues are key contributors to ligand recognition and binding stability within the active site. Notably, CID-87014 displayed the most extensive interaction profile among the screened compounds, supporting its potential as a promising inhibitor of the target protein.

### 3.11 Molecular dynamics simulation

Molecular dynamics (MD) simulations provide valuable insights into the structural behavior of molecules by capturing their motion at the atomic level.^69^ This technique is particularly useful for assessing the stability of a ligand when bound to its target protein.^70^ In this study, a 150 ns MD simulation was carried out to analyze the structural dynamics of selected compound–protein complexes, focusing on how effectively the ligands fit within the protein's active site. The stability and interaction profiles were evaluated through multiple parameters, including MolSA, SASA, RMSF, Rg, PSA, intramolecular hydrogen bonding, RMSD, and protein–ligand contact patterns. All reported MD metrics are expressed as mean ± standard error of the mean (SEM) to provide statistical reliability and facilitate comparison (Table 4). Reporting mean values along with SEM provides both the central tendency and the precision of the measurements, indicating how reliably the estimated mean represents the overall dataset.

**Table 4:** Molecular dynamics simulation parameters for the protein–ligand complexes.

Compounds IDs	RMSD (Mean ± SEM) (Å)	RMSF (Mean ± SEM) (Å)	SASA (Mean ± SEM) (Å^2^)	Rg (Mean ± SEM) (Å)	MolSA (SEM) (Å^2^)	PSA (Mean ± SEM) (Å^2^)
Apo	2.11 ± 0.01	1.05 ± 0.03	–	–	–	–
CID-3220	3.08 ± 0.03	1.02 ± 0.04	44.19 ± 0.65	3.27 ± 0	235.37 ± 0.04	198.71 ± 0.07
CID-442877	2.71 ± 0.02	1.2 ± 0.05	52.49 ± 0.59	2.82 ± 0	196.68 ± 0.04	25.63 ± 0.04
CID-87014	1.77 ± 0.01	1.08 ± 0.04	186.5 ± 0.63	3.21 ± 0	224.94 ± 0.04	121.26 ± 0.07
Control (Y3M)	1.98 ± 0.01	1.13 ± 0.04	185.48 ± 1.18	4.32 ± 0.01	404.26 ± 0.27	127.68 ± 0.21

#### 3.11.1 RMSD analysis of 4UYN and top ligands

**Fig. 9:**
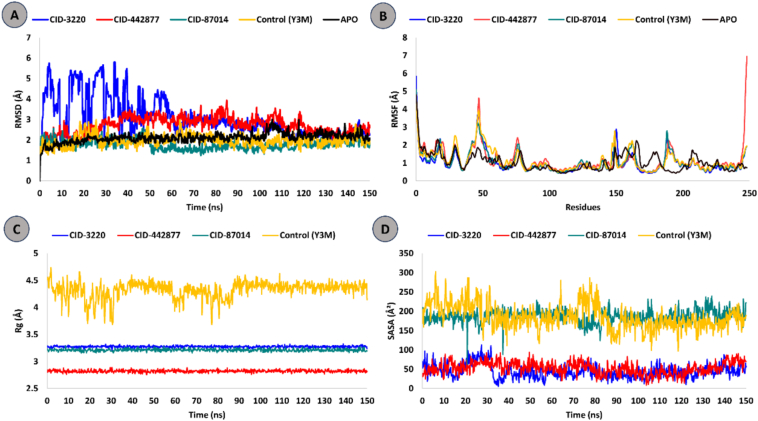
Comparative Analysis of Stability, Flexibility, Compactness, and Solvent Exposure of Protein–Ligand Complexes and the Control System During 150 ns Molecular Dynamics Simulations, Represented by RMSD (A), RMSF (B), Radius of Gyration (Rg) (C), and Solvent-Accessible Surface Area (SASA) (D) Profiles.

The structural stability of the protein and protein–ligand complexes was evaluated by analyzing the RMSD trajectories throughout the molecular dynamics simulation period (Fig. 9A). The apo protein exhibited an average RMSD value of 2.11 ± 0.01 Å, indicating overall structural stability during the simulation. Among the ligand-bound complexes, CID-3220 showed the highest average RMSD (3.08 ± 0.03 Å), followed by CID-442877 (2.71 ± 0.02 Å), suggesting relatively greater conformational deviations from the initial structure. In contrast, CID-87014 displayed the lowest RMSD value (1.77 ± 0.01 Å), indicating excellent structural stability and minimal fluctuations throughout the simulation. The control complex (Y3M) exhibited an average RMSD of 1.98 ± 0.01 Å and remained stable over the simulation period. Particularly, the CID-87014 complex demonstrated lower RMSD values than the control, indicating comparable or marginally improved structural stability. Although the CID-3220 and CID-442877 complexes exhibited higher RMSD values relative to both the apo protein and control complex, their RMSD values remained within an acceptable range, indicating that the complexes retained overall structural integrity during the simulation. These findings suggest that CID-87014 formed the most stable complex with the target protein among the compounds evaluated.

#### 3.11.2 RMSF analysis of 4UYN and top ligands

The Root Mean Square Fluctuation (RMSF) is a useful parameter for examining local flexibility within a protein. It reflects how much a particular atom deviates from its reference position by calculating the average fluctuation of that atom across the simulation timeframe.^54^, ^71^ Larger RMSF values are typically associated with enhanced dynamic motion and structural flexibility of the protein during MD simulations.^72^ The apo protein exhibited an average RMSF value of 1.05 ± 0.03 Å. Among the ligand-bound complexes, CID-3220 showed the lowest average RMSF value (1.02 ± 0.04 Å), indicating the least overall residue fluctuation and suggesting a stabilizing effect on protein dynamics. The CID-87014 complex displayed an RMSF value of 1.08 ± 0.04 Å, which was comparable to that of the apo protein and slightly lower than the control complex (1.13 ± 0.04 Å), indicating maintained structural stability throughout the simulation. In contrast, CID-442877 exhibited the highest RMSF value (1.20 ± 0.05 Å), reflecting relatively greater residue mobility compared with the other complexes. However, the differences among the complexes were modest, and all RMSF values remained within a narrow range, suggesting that ligand binding did not induce substantial fluctuations or destabilization of the protein structure. Overall, CID-3220 demonstrated the lowest residue-level flexibility, while CID-87014 also maintained favorable dynamic stability relative to the control complex (Fig. 9B).

#### 3.11.3 Rg analysis of 4UYN and top ligands

The radius of gyration (Rg) is a structural parameter that reflects the degree of compactness in a protein. It is determined as the root mean square distance of the protein's atoms from its center of mass. A lower Rg value generally indicates a more compact, globular structure, while a higher Rg value corresponds to a more extended and flexible conformation.^73^ The average Rg values for the CID-3220, CID-442877, CID-87014, and control (Y3M) complexes were 3.27 ± 0.00 Å, 2.82 ± 0.00 Å, 3.21 ± 0.00 Å, and 4.32 ± 0.01 Å, respectively. Among the investigated compounds, CID-442877 exhibited the lowest Rg value, indicating the highest degree of structural compactness during the simulation period. The CID-87014 and CID-3220 complexes also maintained relatively low Rg values, suggesting stable and compact conformations throughout the 150 ns simulation. In contrast, the control complex showed the highest Rg value, reflecting a comparatively less compact structural arrangement. Furthermore, the negligible SEM values observed for all complexes indicate minimal variation in Rg over time, suggesting that the protein–ligand systems maintained consistent structural compactness throughout the simulation. Overall, the Rg analysis indicates that the investigated compounds, particularly CID-442877, promoted a more compact structural organization than the reference ligand while preserving the overall stability of the complexes (Fig. 9C).

#### 3.11.4 SASA analysis of 4UYN and top ligands

The solvent-accessible surface area (SASA) refers to the portion of a protein's surface that can be reached by surrounding solvent molecules. This parameter is often used to understand structural changes in proteins, as well as their interactions with ligands or other proteins.^74^, ^75^ Higher SASA values reflect increased exposure of the protein surface to solvent, whereas lower values indicate regions that are tightly packed or buried within the protein structure.^76^ The average SASA values for the CID-3220, CID-442877, CID-87014, and control (Y3M) complexes were 44.19 ± 0.65 Å^2^, 52.49 ± 0.59 Å^2^, 186.50 ± 0.63 Å^2^, and 185.48 ± 1.18 Å^2^, respectively. Among the investigated compounds, CID-3220 exhibited the lowest SASA value, followed by CID-442877, suggesting a comparatively more compact structural arrangement and reduced solvent accessibility during the simulation. In contrast, the CID-87014 complex displayed a SASA value very similar to that of the control complex, indicating a comparable degree of solvent exposure and structural organization. Despite the observed differences in SASA values among the complexes, the relatively low SEM values suggest that the solvent-accessible surface areas remained consistent throughout the 150 ns simulation period. Overall, the results indicate that all complexes maintained stable solvent exposure profiles, with CID-3220 and CID-442877 exhibiting greater structural compactness, while CID-87014 showed behavior closely resembling that of the reference ligand (Fig. 9D).

#### 3.11.5 MolSA and PSA analysis of 4UYN and top ligands

The Molecular Surface Area (MolSA) of the protein–ligand complexes was evaluated over the 150 ns molecular dynamics simulation to investigate variations in molecular surface characteristics and structural compactness (**Fig. S3A**). MolSA represents the solvent-excluded surface area of a molecular system and provides insight into its overall geometric organization. The average MolSA values for the CID-3220, CID-442877, CID-87014, and control (Y3M) complexes were 235.37 ± 0.04 Å^2^, 196.68 ± 0.04 Å^2^, 224.94 ± 0.04 Å^2^, and 404.26 ± 0.27 Å^2^, respectively. Among the investigated compounds, CID-442877 exhibited the lowest MolSA value, followed by CID-87014 and CID-3220, indicating comparatively smaller solvent-excluded surface areas than the control complex. In contrast, the control complex displayed a substantially higher MolSA value, reflecting a larger molecular surface area throughout the simulation period. The consistently low SEM values observed for all complexes indicate minimal fluctuations in MolSA during the 150 ns simulation, suggesting stable surface characteristics and the absence of major structural rearrangements. Overall, the lower MolSA values of the investigated compounds compared with the control imply a relatively more compact molecular organization, while the stable MolSA profiles throughout the simulation support the structural integrity of the protein–ligand complexes.

Polar surface area (PSA), determined by the contributions of oxygen and nitrogen atoms,^77^ was tracked to assess the polar surface exposure of protein–ligand complexes throughout the 150 ns simulation (**Fig. S3B**). The average PSA values for the CID-3220, CID-442877, CID-87014, and control (Y3M) complexes were 198.71 ± 0.07 Å^2^, 25.63 ± 0.04 Å^2^, 121.26 ± 0.07 Å^2^, and 127.68 ± 0.21 Å^2^, respectively. Among the investigated compounds, CID-3220 exhibited the highest PSA value, indicating a greater extent of exposed polar surface area during the simulation. In contrast, CID-442877 showed a markedly lower PSA value, suggesting reduced polar surface exposure relative to the other complexes. The PSA value of CID-87014 was comparable to that of the control complex, indicating similar polar surface characteristics and solvent interaction potential. Furthermore, the low SEM values observed across all complexes indicate that the PSA profiles remained highly consistent throughout the 150 ns simulation period. Overall, the results suggest that the investigated compounds differ in their degree of polar surface exposure, with CID-3220 displaying the greatest polar surface accessibility, CID-442877 exhibiting the least, and CID-87014 demonstrating behavior closely comparable to that of the reference ligand.

#### 3.11.6 Analysis of intramolecular bonds

**Fig. 10:**
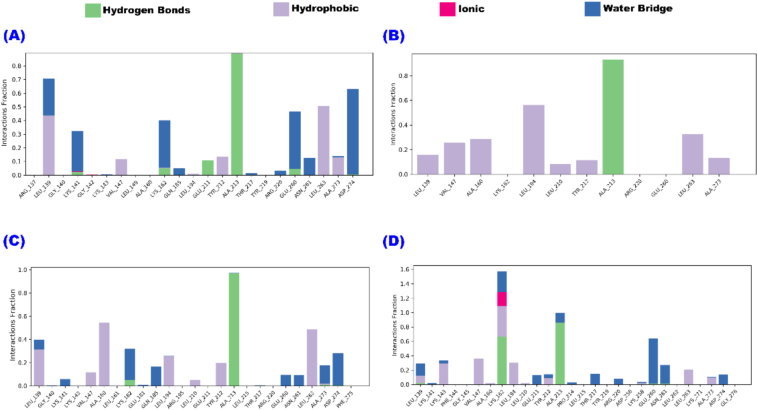
Comparative Interaction Profiles of Candidate Ligands with the Target Protein During 150 ns Molecular Dynamics Simulations. Panels represent the complexes of CID-3220 (A), CID-442877 (B), CID-87014 (C), and the control ligand Y3M (D).

To further investigate the binding stability of the selected ligands within the active site, protein–ligand intermolecular interactions were monitored throughout the 150 ns molecular dynamics simulation using the Simulation Interaction Diagram (SID). The interactions mainly consisted of hydrogen bonds, hydrophobic contacts, water bridges, and ionic interactions (Fig. 10). Among the screened compounds, CID-3220 exhibited a diverse interaction profile characterized by a highly persistent hydrogen bond with Ala213, which remained the dominant interaction throughout the simulation. Additional stabilization was provided by hydrophobic contacts involving Leu139, Val147, Tyr212, and Leu263, together with several water-mediated interactions, particularly with Leu139, Lys162, Glu260, and Asp274, suggesting stable occupancy within the binding pocket. CID-442877 displayed a comparatively compact interaction network, dominated by a strong hydrogen bond with Ala213 and hydrophobic contacts primarily involving Leu194, Val147, Ala160, and Leu263, indicating a well-defined and stable binding mode. Similarly, CID-87014 maintained the most persistent hydrogen-bond interaction with Ala213, accompanied by hydrophobic interactions with Ala160 and Leu263, as well as multiple water-bridge contacts involving Arg137, Lys162, Glu260, Asn260, and Asp274, reflecting strong and sustained ligand stabilization throughout the simulation period. In contrast, the reference ligand Y3M exhibited the most extensive interaction network, involving hydrogen bonds, hydrophobic contacts, water bridges, and ionic interactions. Notably, Lys162 showed the highest overall interaction fraction and was involved in all four interaction types, while Ala213 maintained a strong hydrogen bond. Overall, Ala213 emerged as a key anchoring residue across all ligand–protein complexes, consistently forming persistent hydrogen bonds and highlighting its critical role in maintaining stable ligand binding during the molecular dynamics simulation.

### 3.12 Post simulation MMGBSA analysis

**Fig. 11:**
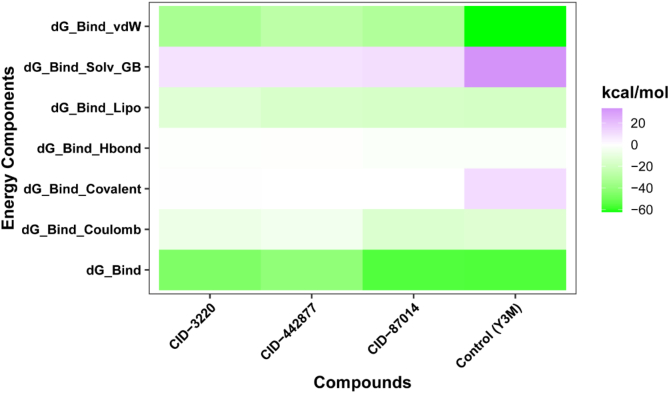
Comparative MMGBSA-based free energy analysis of candidate ligands.

Molecular Mechanics–Generalized Born Surface Area (MM-GBSA) binding free energy analysis helps evaluate how strongly and stably a ligand binds to a protein. In this context, a more negative dG value reflects a higher binding affinity.^78^ The calculated dG_bind values for CID-3220, CID-442877, CID-87014, and the reference ligand (Y3M) were − 46.25, −41.24, −55.73, and − 56.24 kcal/mol, respectively. As more negative binding free energy values indicate stronger binding affinity, CID-87014 exhibited the most favorable binding energy among the investigated compounds and showed a binding affinity comparable to that of the reference ligand. In contrast, CID-442877 displayed the least favorable binding energy, whereas CID-3220 demonstrated an intermediate binding affinity. Analysis of the individual energy components revealed that van der Waals (dG_bind_vdW) and lipophilic (dG_bind_Lipo) interactions were the major favorable contributors to complex formation across all compounds. Notably, CID-87014 showed stronger Coulombic interactions (−15.10 kcal/mol) than CID-3220 and CID-442877, indicating a greater contribution from electrostatic interactions to its binding stability. Hydrogen-bonding interactions contributed modestly to the overall binding energy in all complexes. Conversely, the solvation energy term (dG_bind_Solv_GB) contributed positively to the total free energy, thereby opposing ligand binding. Overall, the MM/GBSA results suggest that CID-87014 possesses the most favorable binding profile among the investigated compounds and exhibits a binding affinity closely comparable to that of the reference ligand, supporting its potential as a promising candidate for further investigation (Fig. 11).

## 4 Discussion

OC remains one of the deadliest gynecologic cancers due to late diagnosis, frequent relapse, and chemoresistance, demanding better treatment options. By integrating transcriptomic profiling, network analysis, and molecular docking, this study identified key therapeutic targets in OC and screened phytochemicals with strong anticancer potential. These computational findings point toward plant-derived candidates with potentially improved predicted safety profiles. This study combined transcriptomic analysis, systems biology, and computational drug design to discover phytochemical-based therapies for OC.

In this study, across the three independent datasets (GSE14407, GSE18520, and GSE26712), we identified a consistent set of commonly dysregulated genes, indicating robust molecular alterations associated with ovarian cancer despite inter-dataset variability. The recurrence of these cDEGs suggests their potential involvement in key molecular mechanisms underlying ovarian cancer initiation and progression. Therefore, these shared genes provide reliable candidates for downstream functional analysis and biomarker discovery. Functional enrichment of the common DEGs suggests that ovarian cancer is largely driven by disruption of pathways controlling proliferation, genomic stability, and cellular movement. Aberrant regulation of cell-cycle and mitotic processes may promote rapid tumor expansion, while migration-related signatures support the invasive and metastatic nature of the disease. Enrichment within nuclear and mitotic compartments further indicates active transcriptional reprogramming and chromosomal instability in tumor cells. In addition, signaling pathways such as PI3K-Akt and p53 imply coordinated effects on survival, apoptosis resistance, and DNA damage response. These findings are consistent with previous studies highlighting the central role of cell-cycle dysregulation and genomic instability in ovarian cancer progression, collectively suggesting biologically relevant mechanisms that may serve as therapeutic targets.^79^ Moreover, the integrated TF–miRNA network indicates that ovarian cancer progression may be regulated through coordinated transcriptional and post-transcriptional mechanisms involving key regulators associated with proliferation, differentiation, and cell-cycle control. Similarly, the identified miRNAs and their coordinated interactions within the regulatory network suggest potential roles in ovarian cancer progression and highlight them as promising biomarkers and therapeutic targets. Then, the PPI network analysis highlights that ovarian cancer–associated genes function in a highly interconnected manner rather than through isolated pathways, emphasizing the cooperative nature of tumor progression. Recurrently ranked hub genes were mainly linked to mitotic control and cell-cycle regulation, reinforcing the importance of uncontrolled proliferation in ovarian cancer pathogenesis. Although several hubs showed strong centrality, therapeutic prioritization required considering not only network importance but also biological specificity and druggability. In this context, AURKA emerged as the most practical candidate because its dysregulation is closely associated with chromosomal instability and malignant progression, while its kinase domain offers a tractable site for targeted inhibition. Although AURKA is not a novel discovery, it is a well-established regulator of cell cycle progression and mitotic control in cancer biology, as identified through extensive literature review.^80^, ^81^, ^82^ Consistent with prior reports linking AURKA to chromosomal instability,^79^ and poor prognosis in ovarian cancer, as well as ongoing efforts to therapeutically target this kinase, our results further corroborate its central role in ovarian tumor progression. Thus, the identification of AURKA in our study reinforces the robustness of the analytical pipeline, as it successfully recapitulates a well-known oncogenic driver in ovarian cancer. Importantly, its consistent dysregulation highlights its potential as a therapeutic target and supports the concept of shared oncogenic mechanisms within ovarian malignancies. Previous studies investigating AURKA inhibition in ovarian and other cancers have primarily focused on synthetic kinase inhibitors and experimental compounds, many of which are associated with toxicity, resistance, or suboptimal pharmacokinetic properties.^83^ In contrast, plant-derived AURKA inhibitors remain comparatively underexplored despite their potential structural diversity and favorable safety profiles. Additionally, AURKA showed strong overexpression at mRNA and protein levels across multiple tumor types using TIMER2.0, GEPIA2, and UALCAN. Correlation profiling revealed cancer-specific variations in AURKA's correlation with individual hub genes. These context-dependent correlations suggest that AURKA may function within distinct regulatory modules depending on tumor type, rather than acting as a uniform driver across all cancers. In immune infiltration analysis negative correlations are strongest and most prevalent in epithelial cancers like breast (BRCA subtypes), kidney (KICH/KIRP), lung (LUAD), and STAD, especially in activated_CIBERSORT−ABS and activated CIBERSORT methods. Positive correlations are strongest in UVM, LUAD and STAD, mainly in resting mast cell CIBERSORT and XCELL modes. These patterns indicate a potential immunomodulatory role of AURKA, where it may influence tumor–immune interactions in a context-dependent manner. These findings suggest that AURKA may suppress mast cell infiltration to promote immune evasion in aggressive tumors, while in certain inflammatory contexts it might instead facilitate mast cell recruitment to support tumor progression. The Macrophage_M0_CIBERSORT method exhibits the strongest positive correlation with AURKA in GBM, and Macrophage_M1_CIBERSORT method exhibits the strongest positive correlation with AURKA in BRCA, suggesting a significant link to tumor progression. Other cancers like PAAD, UVM and KIRC show moderate positive correlations (Macrophage_EPIC), while THYM display negative correlations across nearly all methods, indicating possible distinct immune dynamics. Among all immune infiltration methods, Th2_XCELL uniquely revealed a consistent positive correlation between gene expression and CD4+ T cells across all cancer types (*p* < 0.05), highlighting its potential sensitivity in capturing tumor–immune interactions. The AURKA crystal structure (PDB: 4UYN) was refined for docking and screened against 2046 phytochemicals. Since a lower docking score indicates tighter binding between ligand and protein,^84^ these top 10 compounds showed binding potentials (−8.155 to −7.634 kcal/mol) comparable to the control inhibitor Y3M (−8.449 kcal/mol).

ADMET analysis identified five compounds (CID-12310964, CID-65084, CID-3220, CID-442877, and CID-87014) with favorable pharmacokinetic properties for further investigation. All selected compounds satisfied Lipinski's rule of five,^85^ confirming acceptable drug-likeness. CID-442877 and CID-87014 showed the most favorable profiles with high gastrointestinal absorption, moderate solubility, low polar surface area, and limited CYP450 inhibition, along with easy synthesis. CID-65084 exhibited the best solubility and no CYP inhibition, minimizing drug–drug interaction risk, although its higher polar surface area may limit permeability. In contrast, CID-12310964 and CID-3220 were less favorable due to higher CYP inhibition risks. The control compound Y3M (CID-86278048) exhibited poor pharmacokinetic properties, including low absorption, weak solubility, multiple CYP inhibitions, and challenging synthesis, as reflected by its relatively high Synthetic Accessibility (SA) score, noting that lower SA scores indicate easier synthesis while higher scores correspond to more complex or difficult synthesis.^86^ Overall, CID-442877 and CID-87014 emerged as the most promising candidates based on combined docking and ADME prediction results. The provided compounds exhibit varied toxicity profiles: CIDs 12,310,964, 65084, 442877, and 87,014 show positive AMES toxicity but negative for hERG II inhibition, hepatotoxicity, and skin sensitisation, indicating potential mutagenicity without cardiac, liver, or dermal risks. In contrast, CID 3220 is negative across all tests, suggesting the lowest toxicity. Compared with the control, these compounds showed fewer predicted toxicity liabilities across the evaluated in silico endpoints. Most anticancer drugs like Cyclophosphamide, Cisplatin, Mitomycin C, Doxorubicin and Bleomycin test positive in the AMES assay due to their DNA-damaging mechanisms essential for targeting cancer cells,^87^, ^88^, ^89^, ^90^, ^91^ aligning with the AMES-positive results here for four compounds. This supports their further evaluation as candidates for oncology applications, while their mutagenicity predictions require careful experimental assessment. Further validation through complementary genotoxicity assays, such as the in vitro micronucleus assay, Comet assay (single-cell gel electrophoresis), chromosomal aberration test, mouse lymphoma assay, and in vivo micronucleus test, would be necessary to better characterize their potential mutagenic and DNA-damaging effects. According to the integrated assessment of docking performance, ADME characteristics, toxicity prediction, and pharmacokinetic suitability, CID-3220, CID-442877, and CID-87014 demonstrated comparatively more favorable overall profiles than the other investigated phytochemicals. In contrast, CID-12310964 exhibited predicted inhibition of multiple CYP450 enzymes, which may increase the risk of drug–drug interactions and undesirable pharmacokinetic behavior, thereby reducing its suitability as a potential therapeutic candidate. Although CID-65084 demonstrated excellent solubility and no predicted CYP inhibition, its relatively high polar surface area may negatively influence membrane permeability and oral bioavailability. In contrast, CID-3220 exhibited the most favorable toxicity profile, whereas CID-442877 and CID-87014 displayed a more balanced combination of ADME characteristics and drug-likeness properties. Collectively, these findings suggest that CID-3220, CID-442877, and CID-87014 possess comparatively more favorable pharmacological profiles than the other investigated phytochemicals. Consistent with the ADMET findings, the DFT analysis further supported the prioritization of CID-3220, CID-442877, and CID-87014. While CID-12310964 exhibited high electronic reactivity and CID-65084 demonstrated good stability, their overall profiles were less favorable when electronic properties were considered alongside pharmacokinetic suitability and toxicity predictions.

Moreover, the interaction results indicated that all selected phytochemicals bind within the same active-site pocket as the reference ligand and share several key interactions with residues involved in ligand recognition. The consistent involvement of LEU263, VAL147, ALA273, and ASP274 across multiple ligand–protein complexes highlight their potential importance in maintaining binding stability within the active site. Among the screened compounds, CID-87014 exhibited the most extensive interaction network, suggesting a favorable binding orientation and stronger stabilization within the binding pocket. These findings support its prioritization for subsequent molecular dynamics simulations and binding free-energy analyses.

The molecular dynamics simulation and post-simulation MM/GBSA analyses collectively demonstrated that the selected compounds maintained stable interactions with the target protein throughout the 150 ns simulation period. RMSD analysis revealed that CID-87014 exhibited the lowest conformational deviation among the ligand-bound complexes, indicating superior structural stability compared with both the investigated compounds and the reference ligand. This observation was further supported by its favorable RMSF profile, suggesting that ligand binding did not induce excessive residue-level fluctuations. Although CID-442877 displayed the highest RMSF value, the differences among the complexes were relatively small, indicating that all systems remained structurally stable during the simulation. The Rg, SASA, and MolSA analyses further confirmed the maintenance of compact protein conformations, with CID-442877 showing the greatest structural compactness, while CID-87014 exhibited solvent exposure characteristics comparable to the reference ligand. Interaction profiling revealed that Ala213 served as a crucial anchoring residue across all complexes, forming persistent hydrogen-bond interactions that contributed to binding stability. Importantly, MM/GBSA calculations identified CID-87014 as the most promising candidate among the screened compounds, exhibiting a binding free energy very close to that of the reference ligand. The strong contribution of van der Waals, lipophilic, and electrostatic interactions to its binding affinity further supports its potential as a stable and effective inhibitor. Based on the integrative analyses, CID-87014 emerged as the most promising phytochemical lead against AURKA in OC, showing consistent performance across docking, pharmacokinetics, molecular dynamics, and quantum mechanical evaluations. Notably, this study addresses a key gap in current research by highlighting plant-derived AURKA inhibitors, which remain underexplored compared to synthetic compounds. Taken together, these findings highlight CID-87014 as the strongest lead compound for further experimental validation.

## 5 Limitations of the study

This study is primarily based on in silico analyses, which, despite being comprehensive, cannot fully capture complex biological systems and therefore require further in vitro and in vivo validation. Although three independent GEO datasets were integrated to improve robustness, variations in platforms and experimental conditions may still introduce some bias. Hub gene identification also depends on network-based methods and algorithm selection, even though multiple approaches were applied to enhance reliability. In addition, this work uses a focused phytochemical library, making it a proof-of-concept screening; exploring larger and more diverse compound sets may reveal stronger candidates. ADMET and toxicity predictions, including the observation that some compounds showed AMES positivity, are based on computational models and may not fully reflect real biological responses. Furthermore, the 150 ns molecular dynamics simulation provides initial insights into stability but may not capture long-term conformational changes. Overall, these findings are preliminary and need experimental validation to confirm their therapeutic potential.

## 6 Conclusion

In this study, an integrative in silico approach combining transcriptomic analysis, network biology, and molecular modeling identified AURKA as a key therapeutic target in ovarian cancer. Although the synthetic control exhibited stronger initial docking affinity, CID-87014 demonstrated superior integrated performance across molecular dynamics stability, ADMET suitability, and quantum descriptors, identifying it as the most promising natural AURKA inhibitor candidate among the screened phytochemicals. However, as these findings are based solely on computational analyses and considering its predicted AMES positivity, CID-87014 should be regarded as a potential candidate requiring cautious experimental validation. To validate its therapeutic relevance, future studies should include in vitro cytotoxicity assays in ovarian cancer cell lines such as SKOV3 and OVCAR3, followed by AURKA kinase inhibition assays and mechanistic analyses including apoptosis and cell-cycle evaluation. Promising results may further be validated in in vivo xenograft models. Therefore, this study highlights the potential of natural product–based screening approaches for identifying candidate molecules targeting key oncogenic drivers in gynecologic cancers, while emphasizing the need for experimental validation to confirm their clinical relevance.

## CRediT authorship contribution statement

**Hriddhi Sarker:** Writing – original draft, Visualization, Validation, Software, Resources, Methodology, Investigation, Formal analysis, Data curation, Conceptualization. **Md. Ahad Ali:** Writing – review & editing, Writing – original draft, Visualization, Validation, Supervision, Software, Resources, Methodology, Investigation, Formal analysis, Data curation, Conceptualization. **Md Fakhrul Islam:** Visualization, Investigation, Formal analysis, Data curation. **Enam Ahmed:** Software, Resources, Formal analysis, Data curation. **Amlan Ganguly:** Writing – review & editing, Supervision, Project administration. **Md. Nazmul Hasan Zilani:** Visualization, Software, Resources, Formal analysis.

## Ethics approval and consent to participate

This research involves no animal model. So, this is not applicable to this context.

## Funding

No funding was received by the author(s) for conducting the research, writing, or publishing this work.

## Declaration of competing interest

We can confirm that the work entitled “*Integrative Multi-Omics and Network Pharmacology Reveal Natural Therapeutics as Anti-Cancer Agents Targeting AURKA for Ovarian Cancer Treatment*” is an original work and neither this nor any similar manuscript, in whole or in part, is under consideration, in a press, published, or reported elsewhere. All co-authors have seen and agreed with the contents of the manuscript. We would like to declare that we have no any conflict of interest for this work. Anyone can use any information from our manuscript.
