# Supplementary material for: Integrative multi-transcriptomics and network pharmacology reveal natural therapeutics as anti-cancer agents targeting AURKA for ovarian cancer treatment
Source: J Genet Eng Biotechnol. 2026 Jun 20;24(3):100745. doi: 10.1016/j.jgeb.2026.100745 (PMC13314747; doi:10.1016/j.jgeb.2026.100745)
Supplement: Supplementary file 2 — Supplementary material 2 [file mmc2.docx]

**
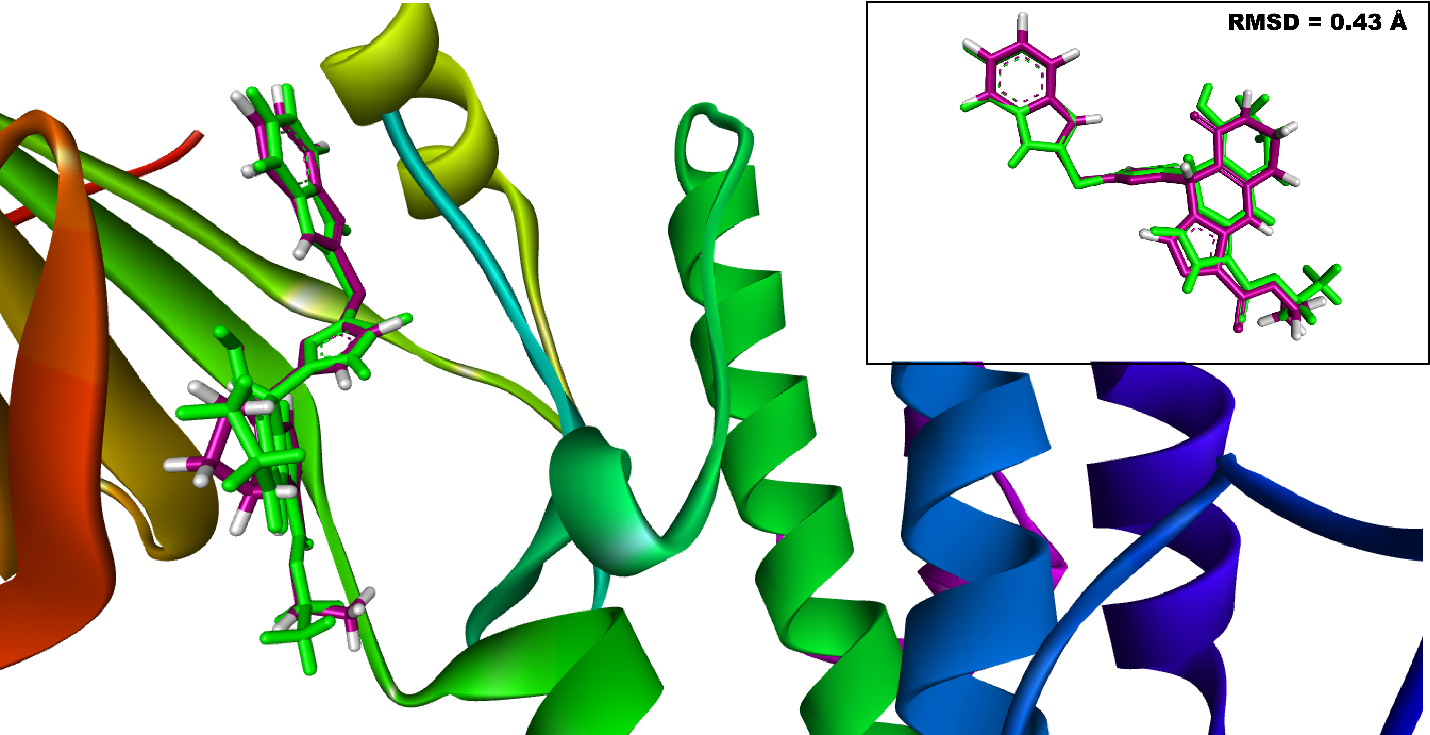
**

**Figure S1.** Validation of the docking protocol by redocking of the co-crystallized ligand (Y3M) into the active site of 4UYN, showing superimposition of the docked pose (green) with the native crystallographic pose (magenta), with an RMSD of 0.43 Å.


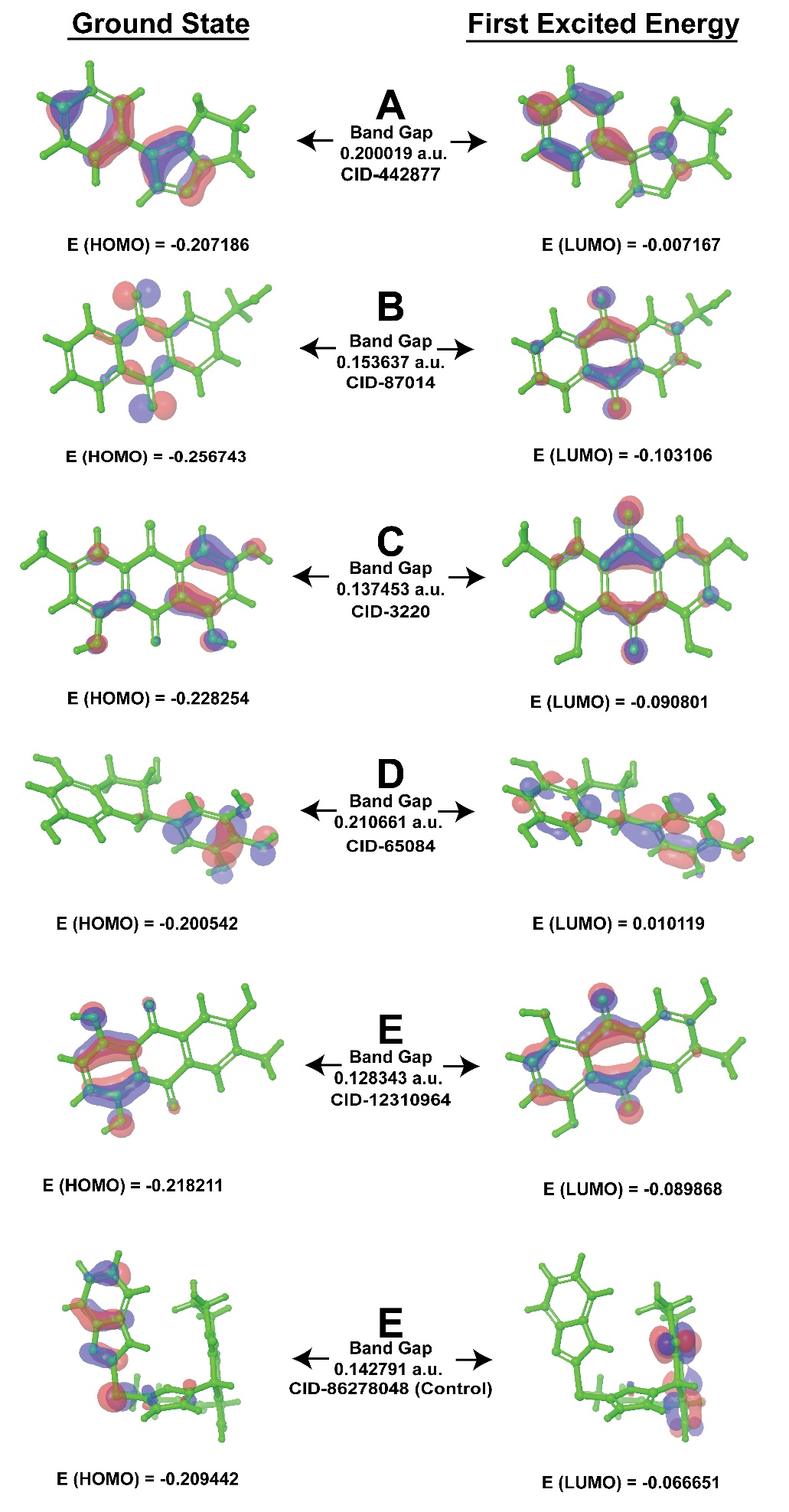


**Figure S2**. Frontier molecular orbital (FMO) distributions (HOMO and LUMO) of the selected ligands showing electron density patterns associated with chemical reactivity and stability.


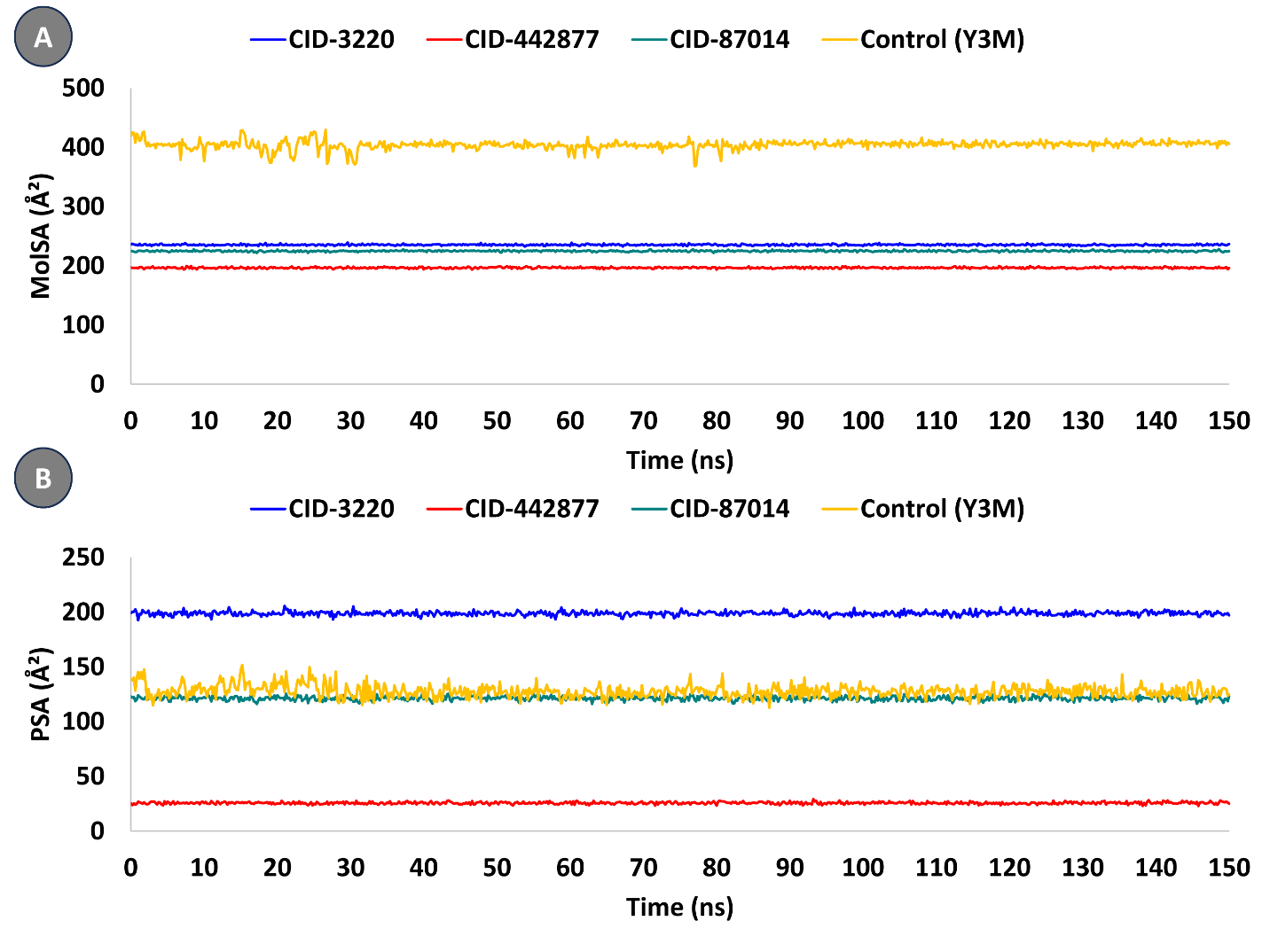


**Figure S3.** Comparative analysis of (A) Molecular Surface Area (MolSA) and (B) Polar Surface Area (PSA) of candidate ligands (CID-12310964, CID-65084, CID-3220, CID-442877, CID-87014) in complex with the target protein, with CID-86278048 serving as the control, across 100 ns MD simulation.
